# Effects of Tobacco Taxation and Pricing on Smoking Behavior in High Risk Populations: A Knowledge Synthesis

**DOI:** 10.3390/ijerph8114118

**Published:** 2011-10-26

**Authors:** Pearl Bader, David Boisclair, Roberta Ferrence

**Affiliations:** 1 Consultants in Behavior Change, 250 Heath Street East, Toronto, ON M4T 1T2, Canada; 2 Consultant in Economics and Public Health, 5946 de Bordeaux, Montreal, QC H2G 2R7, Canada; E-Mail: davidboisclair@yahoo.ca; 3 Ontario Tobacco Research Unit, Dalla Lana School of Public Health, University of Toronto, 33 Russell Street, Toronto, ON M5S 2S1, Canada; E-Mail: roberta_ferrence@camh.net

**Keywords:** tobacco taxation and pricing, high-risk subpopulations, public health policy, smoking cessation

## Abstract

Tobacco taxation is an essential component of a comprehensive tobacco control strategy. However, to fully realize the benefits it is vital to understand the impact of increased taxes among high-risk subpopulations. Are they influenced to the same extent as the general population? Do they need additional measures to influence smoking behavior? The objectives of this study were to synthesize the evidence regarding differential effects of taxation and price on smoking in: youth, young adults, persons of low socio-economic status, with dual diagnoses, heavy/long-term smokers, and Aboriginal people. Using a better practices approach, a knowledge synthesis was conducted using a systematic review of the literature and an expert advisory panel. Experts were involved in developing the study plan, discussing findings, developing policy recommendations, and identifying priorities for future research. Most studies found that raising cigarette prices through increased taxes is a highly effective measure for reducing smoking among youth, young adults, and persons of low socioeconomic status. However, there is a striking lack of evidence about the impact of increasing cigarette prices on smoking behavior in heavy/long-term smokers, persons with a dual diagnosis and Aboriginals. Given their high prevalence of smoking, urgent attention is needed to develop effective policies for the six subpopulations reviewed. These findings will be of value to policy-makers and researchers in their efforts to improve the effectiveness of tobacco control measures, especially with subpopulations at most risk. Although specific studies are needed, tobacco taxation is a key policy measure for driving success.

## 1. Introduction

If it were totally up to me, I would raise the cigarette tax so high the revenues from it would go to zero.

– Michael Bloomberg (New York mayor)

Significant gains have been made in reducing the prevalence of smoking for the general population in North America and other high-income countries. However, smoking rates remain high for some subpopulations. Responses to tobacco control interventions and pathways to change in smoking behavior can vary substantially among subgroups of smokers. Even for those who benefit from these measures, they do not gain equally and major disparities exist.

Tobacco taxation, passed on to consumers in the form of higher cigarette prices, has been recognized as one of the most effective population-based strategies for decreasing smoking and its adverse health consequences [1–4]. On average, a price increase of 10% on a pack of cigarettes would reduce demand for cigarettes by about 4% for the general adult population in high income countries [4]. Tobacco taxes can benefit smokers who quit, reduce the overall consumption of tobacco, and put smoking cessation on the radar of those who continue to smoke. Increased taxes also have a positive impact on non-smokers by reducing their exposure to second-hand smoke. However, much less is known about the impact of taxation on specific subgroups. Are they influenced to the same extent as the general population? Do they need additional measures and initiatives to reduce smoking?

Using a better practices approach to knowledge synthesis, this study illustrates the value of using two complementary approaches (systematic review of literature and expert opinion) in an area of high importance but with varying degrees of empirical studies across subpopulations. The Better Practices Model, developed by the Canadian Tobacco Control Research Initiative [5], provides a structure for integrating variable, but complementary, sources of information which can lead to enhanced understanding of chronic diseases. Integral to this approach is the belief that “good solutions to complex problems draw upon both science and experience.” Broad sources of information (e.g., peer-reviewed studies, grey literature, expert opinion) are synthesized to increase knowledge of a particular topic.

We systematically reviewed and synthesized evidence regarding the effects of tobacco taxation and pricing (tobacco taxation and pricing hereafter referred to as “price”) on smoking behavior in six high-risk subpopulations. They were selected based on continued high rates of smoking (Table 1) and greater risk for the health consequences of smoking (note: these are not always distinct categories and there may be considerable overlap with some of these subpopulations. For example, heavy and long-term smoking is characteristic of smokers with dual diagnoses; low socioeconomic status is more common among Aboriginals, dual diagnosis individuals, and heavy smokers).

### 1.1. Youth (<19 years)

Youth (<19 years) are a critical focus for tobacco control policy. Because most adult smokers report smoking onset before the age of 20 years [6–8], if one can reach adulthood without smoking, then the probability of smoking onset is greatly reduced. Although youth continue to smoke, O’Loughlin and colleagues [9] found that 70% of teens express a desire to quit. However, only 19% making a quit attempt remained smoke-free for 12 months or more by the end of the five-year study. Because cessation strategies have not been very effective for youth populations, the research suggests that more needs to be done in terms of legislation, programming and taxation [10].

### 1.2. Young Adults (18–24 years)

Young Adults (18–24 years) continue to smoke at high rates, despite strong public awareness of the health hazards [11]. This developmental period is a time of risk for both initiation of smoking and progression to higher levels [12]. Moreover, smoking among young adults is predictive of smoking in later adulthood. While smoking rates have decreased over the past twenty years for both adults and teens, rates for young adults aged 18 to 24 years have not substantially changed in most high-income countries.

### 1.3. Low Socio-Economic Status (SES)

Smoking is strongly linked to social and economic status and is a significant contributor to inequalities in health. Smoking rates in high income countries are highest among those who have had the least education and are in the lowest socioeconomic groups [13,14].

### 1.4. Dual Diagnosis

Smokers who are diagnosed with mental health and/or non-nicotine substance abuse disorders are disproportionately affected by tobacco dependence. In North America, five to 10 percent of the population has a diagnosable mental illness [15]. Yet, they carry almost half the burden of Canadian and US tobacco consumption, smoking approximately 40% of all cigarettes consumed [15–18].

### 1.5. Heavy and/or Long-Term Smokers

Heavy and/or Long-term smokers are at greater risk for the health consequences of smoking. Both intensity and duration of smoking from onset to cessation have a strong positive association with morbidity and mortality [19]. There is not a consistent definition of either “heavy smokers” or “long-term smokers” in the literature. Generally, studies describe heavy smokers as those who smoke more than 15 cigarettes per day [20], although some define “heavy” by 25 cigarettes per day [21,22]. A measure of “long-term” smokers was not found in the literature we reviewed.

### 1.6. Aboriginal People

Aboriginal people in North America have substantially higher rates of smoking than the general population (Table 1). These rates have changed very little in the past 25 years. Availability of inexpensive cigarettes (in North America, access to inexpensive cigarettes is due to tax-exemption) has been cited as a major contributing factor, exacerbated by relative poor socioeconomic status, lack of access to quality health care, poor physical infrastructure and environmental factors [23–26].

The aim of this study was to determine the differential effects of tobacco taxation and price on six subpopulations compared to the general population, primarily in high income countries.

- *Main Effects*: Do subpopulations respond differently than the general population to changes in tobacco taxation and pricing?
- *Synergistic Effects*: What are the interactive effects between taxation and other tobacco control policies among the subpopulations under review?
- *Inadvertent Effects*: Do subpopulations adopt price minimization strategies (e.g., switching to discount brands, smoking more of a cigarette, contraband) in response to increased cigarette prices?

This article focuses primarily on high-income countries. We present an overview of the main findings and key recommendations. A detailed description of the methods and results is presented in the background report [27].

## 2. Methods

### 2.1. Systematic Review

#### Search Strategy

An extensive search was conducted to identify relevant studies, both published and unpublished, on the impact of price on smoking behavior of the six subpopulations. We used the following sources published from 1975 to November 2010: electronic bibliographic databases (all EBM Reviews, EconLit, Embase, Medline, OTRULIB, PAIS Intl, PolicyFile, PsychInfo, Scopus, Web of Science), key journals in tobacco control and health economics, reference lists from retrieved articles, electronic mailing lists, and economic working papers and other unpublished works recommended by the authors, expert panel members, and colleagues in the field. Members of the expert panel reviewed the final reference list for completeness.

#### Study Selection and Inclusion Criteria

Two independent reviewers assessed titles and abstracts for relevance and inclusion. Disagreements were resolved through discussion. Included studies had a primary focus on the impact of price on smoking initiation, cessation, prevalence, or consumption. Some studies were included because they provided pertinent information regarding the impact of price on the general population or contained important background on the subpopulations.

#### Data Extraction and Quality Assessment

Citations were screened using the above criteria and full texts of all citations considered relevant to the study were obtained. To provide consistent coding of responses, data were extracted using forms adapted from Bader *et al*. [11], which included information on: population subgroup, study design/methods, outcome measures, results, conclusions and recommendations. A notes section for a descriptive summary and limitations of the study was also included. A systematic approach to citation management (Excel) was used to manage the review.

#### Quality Assessment

Each study was rated by one reviewer, and checked by the other, to assess the strength of evidence. Quality was assessed using a checklist adapted from Bader *et al*. [11] and the Effective Public Health Practice Project Quality Assessment Tool [33]. Studies were rated as *Strong, Moderate,* or *Weak* by summing across the individual responses coded as *Strong (2), Moderate (1)* or *Weak* (0) in the Quality Assessment Form and computing a total score (maximum = 2 times # items). A study was rated Strong if it achieved a score of at least 75% of the maximum score, Moderate with 50–75%, and Weak with 50% or less.

#### Data Analysis and Synthesis

Results were analyzed focusing on the question: *Are “specific subpopulation” more responsive to price of cigarettes than the general population?* For Youth and Young Adults, studies were analyzed according to an additional question: *Are there differential responses to price of cigarettes according to various dimensions of smoking behavior—that is, by initiation, cessation, participation, consumption, or transitions to different stages of smoking uptake?* For Low SES, studies were analyzed according to an additional question, *If low socioeconomic populations are more price-responsive, are they sufficiently more price-responsive to counter any adverse effects of increased taxation?* (If populations of low socioeconomic status cut their consumption more than the general population in response to higher prices, do they do so enough to offset their price-minimizing behaviors, such as switching to cheaper or contraband cigarettes, or smoking each cigarette more completely).

### 2.2. Expert Panel

An integral component of this Knowledge Synthesis was a comprehensive evaluation of evidence that included expert knowledge and advice. Experts were identified by a search of the literature for their publications, key note speakers on tobacco taxation at conferences, and recommendations of the authors and other colleagues. The 12 selected experts from Canada, the US, and Australia included: health economists, researchers, epidemiologists, a policy analyst, a psychiatrist and an addiction medicine physician (see Full Report, [27]). The Expert Panel met twice:

1. *Initial Phase*: to obtain feedback on the study plan;
2. *Analysis Phase*: to discuss findings, develop policy recommendations, and identify priorities for future research based on gaps in literature.

## 3. Results

### 3.1. Main Effects

The majority of studies (67) focused on the impact of increased price on youth. In comparison, 19 studies were identified for young adults, 25 for persons with low socio-economic status, three for persons with a dual diagnosis, one for heavy and/or long-term smokers and two for Aboriginal people (7 studies examined both youth and young adults, 1 both youth and low SES, for a total of 108 discrete studies).

There was strong evidence that raising cigarette prices through increased taxes is a more effective tobacco control policy measure for reducing smoking behavior among youth, young adults, and persons of low socioeconomic status, compared to the general population. In contrast, there was a lack of evidence about the impact of price on smoking behavior in persons with a dual diagnosis, heavy and/or long-term smokers, and Aboriginal people.

#### Youth

Sixty-seven studies (57 published; 10 unpublished) met our selection criteria. Quality ratings for all studies were strong or moderate. Most studies were conducted in the US, six were Canadian, and eight were international (Australia, France, Ireland, Spain, Sweden, UK). Because youth smoking is mainly a function of initiation and transitions to higher levels of smoking uptake, and to a lesser extent of cessation, it is essential to understand how increased cigarette prices specifically affect youth smoking behavior. Do higher cigarette prices encourage existing youth smokers to quit? Do higher prices deter nonsmokers from starting? A few studies also posed the question, ‘Do higher cigarette prices deter youth smokers from transitioning from lower to higher stages of smoking uptake’? Twenty-one studies examined the impact of increased cigarette prices on initiation [34–54], seven on initiation and cessation [42,43,48–52], three on cessation alone [38,55,56], five on progression to different stages of smoking uptake [37,55,57–59], and 31 on participation and/or consumption [55,60–89]. Table 2 summarizes the results (some studies may appear more than once if they include findings for different dimensions). Studies examining the effects of increased price on youth generally found that they are two to three times more price-responsive than the general population, although price elasticity estimates vary across studies. The consensus is that increased prices decrease both smoking participation and consumption of cigarettes.

However, the impact of increased price on smoking initiation is less clear. Of 22 studies on the role of price in preventing smoking initiation, seven found that increased price prevents smoking onset [35,43,48,50,52–54], nine found that it does not [38–40,42,43,47,49,51,54], and six found that price prevents initiation in some cases [34,36,37,44–46].

Many studies recognize that youth are not a homogeneous population. The effects of price on smoking behavior depend on age, gender, income, peer and family influences, school status (high school student *vs.* dropout), and broader context (e.g., state sentiment). Twelve studies found differential responses to price by age—that is, younger teens (more likely to be experimental smokers) are not price-responsive, while older teens (more regular smokers) are responsive to price [44,46,55,57,59,71,73–76,84,90]. Explanations include the differing levels of addiction between experimental and regular smoking as well as sources from which youth acquire cigarettes—younger teens at lower levels of smoking intensity “borrowing” *versus* older teens at higher levels purchasing cigarettes. Eleven studies that observed gender differences had mixed results [35–37,42,45,48,52,65,71,77,84]. Only six studies included peer and/or family influences in their analysis [34,43,54,81,86,91].

#### Young Adults

Nineteen studies (16 published; three unpublished) focused on the impact of price on young adults. Quality ratings were strong or moderate. Sixteen studies were conducted in the US, one in Canada, and the other a meta-analysis of international studies. Table 3 summarizes the results (some studies may appear more than once if they include findings for different dimensions).

Eleven studies estimated elasticities for price-responsiveness for participation and/or consumption [7,42,55,60,64,71,92–96], eight studies attempted to discern whether decreases in smoking prevalence are due to smoking initiation or smoking cessation [38,42,49,95,97–100], and one investigated the impact of price on progression to different intensities of smoking behavior [98]. While most studies found that increased prices result in reductions in smoking behavior, the magnitude of the effect tends to be smaller than for youth. The majority of studies found that price is inversely related to both smoking participation and consumption. Price has an impact on encouraging cessation, but as with youth, the impact of price on smoking initiation is less clear. One study that explored transitions to higher levels of smoking uptake [98] found that all three transitions (from no daily smoking to 1/2–1+ packs/day) were responsive to price changes.

#### Low SES

The majority of studies (rated strong or moderate) reported significant smoking participation and consumption effects for low income, low education populations. Twenty-four studies (22 published; two unpublished) met selection criteria. Nineteen published and two unpublished studies were rated as strong or moderate. Studies were conducted in Canada, the US, the UK, other European countries, New Zealand, China/Russia and Mexico. Twelve studies found that persons of low socioeconomic status are more responsive to price than the general population [19,52,96,101–109]. Five indicated that low SES groups have the same responsiveness to price as the general population, that is, increased price appears to benefit all socioeconomic groups equally in terms of reducing both smoking participation and consumption [13,110–113].

A central concern regarding the impact of increased taxes of cigarettes on low socioeconomic status groups is whether or not such a tax is equitable. It has been argued that cigarette taxes are a regressive tax on the poor. A tax is regressive if lower incomes are taxed proportionally more than higher incomes. Therefore, tobacco taxes are regressive in percentage terms, as lower income individuals devote a higher percentage of their income to paying the tobacco tax than do higher income individuals. In addition, because people of lower socioeconomic status (SES) have higher smoking rates, they pay more tobacco tax per capita than those with higher incomes [114].

However, some argue that increasing cigarette taxes is not regressive if it results in differential smoking behavior change—*i.e.*, quitting smoking or reducing consumption of cigarettes at higher rates than the general population. Some propose that increasing tobacco taxes is actually progressive at the population level because of the potentially greater accrued health benefits of reduced smoking [115]. This point of view is still contentious among economists, however, and some estimate that for most intents and purposes, tobacco tax *increases* are also regressive even at the population level [101].

While there are numerous studies that support the effectiveness of increasing prices, most declare that equity implications need to be paramount. Even studies that support increased taxes underscore the need to implement policies or measures to assist those who continue to smoke, especially for those smokers who do not quit or reduce smoking in response to increased taxes and who, as a result, may suffer from financial hardship [116,117]. In other words, increased prices need to be accompanied by strategies to mitigate any adverse consequences of such taxes to low SES populations.

#### Dual Diagnosis

Three studies examined the impact of price on populations with a dual diagnosis. Ong *et al*. [16] found that increased price had a significant effect on smoking participation for smokers with drug or mental disorders, but not for those with alcohol dependence. Saffer and Dave [17] found both smokers with mental disorders and those without were similarly price-responsive. Tekin *et al*. [118], who examined adolescents (grades 7–12), found that adolescent smokers with emotional or behavioral problems were at least as responsive to price as those without such problems.

#### Heavy and/or Long-Term Smokers

Only one study specifically examined the effects of price on the likelihood of quitting smoking for individuals with different smoking intensities, including heavy smokers [119]. Of three tobacco policies investigated (taxation, clean air restrictions, and media/comprehensive campaigns), higher prices had the greatest association with making a quit attempt in the past year, but price was not related to the likelihood of remaining abstinent for three or more months. This study did not look at the impact of this policy on duration of smoking.

#### Aboriginal Persons

Only two studies examined the impact of price on smoking behavior. A Canadian study by Wardman and Khan [24] is a commentary on the high smoking prevalence among Registered Indians, rather than an empirical study. It mentions two reserves that implemented a seven percent tax on tobacco products but found no significant decreases in smoking behavior. Another recent unpublished paper by Matheson [26] analyzes the effect of price on adult smoking behavior in Canada’s Aboriginal communities, distinguishing between direct (individual response to price increase) and indirect effects (the influence of price on an individual through changes in community smoking behavior). Findings indicate that price alone is not effective in reducing smoking (a 10% price increase decreases overall smoking by 0.73%) in the aboriginal communities examined. However, the indirect effect doubles the price elasticity over the direct effect alone.

### 3.2. Synergistic Effects

Although some studies examined the independent effects of tobacco control policies in addition to price, few looked at the synergistic effects or interactions between these policies. Clearly, it can be complicated to separate the effects of various policies when several are in place at the same time. However, it is critical to understand their main and interactive effects for designing interventions that will improve the effectiveness of tobacco control programs.

Only one study examined the interactions among tobacco control policies for youth [87], although 22 (of 67 studies) (Table 7a in [27]) incorporated one or more other tobacco control policies in their analysis. The two most commonly reviewed policies were: Clean Indoor Air laws (restrictions on smoking in public places, private worksites, and high schools) and Youth Access laws (e.g., limits on availability of tobacco products to youth, warning signs at point of sale, and bans on vending machine sales).

While many studies on Young Adults (10 of 18) examined the impact of various tobacco control policies in addition to price, findings related to synergistic effects among policies were quite general. For example, several studies recommended that price increases should be combined with a comprehensive tobacco control program for maximum effectiveness, but did not provide evidence regarding specific contributions from individual policies or combinations of policies.

Only three studies investigated the synergistic effects of different tobacco control policies on low SES populations [13,108,120]. No data were found on the effects of taxation policies and other tobacco control policies on populations with dual diagnosis, heavy and/or long-term smokers, and Aboriginal smokers.

### 3.3. Inadvertent Effects

Few studies in this review evaluated the potential unintended consequences of increased cigarette taxation, such as compensatory smoking behavior or greater use of contraband cigarettes. Indeed, it is crucial to understand the extent to which compensatory smoking behavior or use of contraband cigarettes may alter the intended impact of public health policies so that interventions can be designed with greater effectiveness.

Inadvertent effects of price increases were discussed in only one of the Youth studies [56] and not in any Young Adult studies. Two studies found that increased price resulted in greater demand for smuggled cigarettes among low SES smokers [107,113]. Similarly, Taylor *et al*. [121] found that heavy smokers are particularly likely to purchase contraband cigarettes. One study found relatively high rates of illicit cigarette consumption in three psychiatric populations in Toronto [122]. Commonly cited inadvertent effects of tax-free tobacco products in Aboriginal communities included the increase of smuggling activities and “down the road” sales of on-reserve products—*i.e.*, tobacco products purchased in tax-free communities and sold to residents of communities with taxes [23,24].

## 4. Discussion

Assessing the Main Effects of population strategies, such as tobacco taxation and pricing, on high-risk subpopulations is important for understanding the reach and effectiveness of such strategies. Increased tobacco taxes, passed on to consumers in the form of higher cigarette prices, provide an economic disincentive to those who smoke or may be contemplating smoking. Indeed, evidence from this knowledge synthesis strongly supports increasing cigarette prices through tobacco taxation as a powerful strategy for achieving major reductions in smoking among some, but not all, high-risk populations. This is a highly effective policy tool for reducing smoking participation and consumption among youth, young adults and persons of low socioeconomic status. In contrast, major gaps exist in our knowledge about the impact of price on persons diagnosed with mental health or non-nicotine substance abuse disorders, heavy and/or long-term smokers, and Aboriginal people.

Raising cigarette prices is an effective tobacco control policy in reducing smoking among youth. While most studies of young adults found that increased prices also result in reductions in smoking behavior, the magnitude tends to be smaller than for youth. Chaloupka and Pacula [64] argue that because tobacco is an addictive substance, response to increased prices will occur more slowly than for non-addictive goods; therefore long-term gains may be larger than short-term gains. The impact of increased price on smoking initiation among youth and young adults is less clear. Also, differential responses to price by youth and young adults were associated with other determinants, including age (younger *vs*. older teens), gender, income, school status, and peer and family influences.

The majority of studies found that persons of low socioeconomic status are more responsive to price than the general population. This indicates that increased price has the potential to benefitdisadvantaged groups and thereby contribute to reducing health inequalities. The issue of whether or not cigarette taxes are a regressive tax on the poor remains contentious. However, many studies strongly agree on the importance of addressing poverty and social disparities for those who continue to smoke. In other words, increased price needs to be accompanied by strategies to mitigate adverse consequences of such taxes to low SES populations.

The question remains unanswered about whether smokers with a dual diagnosis, heavy and/or long-term smokers, and Aboriginal smokers respond differently than the general population to changes in price. Since these subpopulations have especially high rates of smoking, there is a pressing need for research on effective policy measures to reduce their smoking.

Although some studies examined the independent effects of tobacco control policies in addition to price, few looked at the synergistic effects or interactions between these policies. It is complicated to separate the effects of policies when several are in place at the same time. However, it is critical to understand their main and interactive effects in order to design interventions that will improve the effectiveness of tobacco control programs. Which specific policy measures work best and in which contexts? Which combinations of policies are most effective in influencing smoking behavior in the six subpopulations examined in this study? The answers to these questions underscore the need for further research.

Few studies evaluated inadvertent effects and potential unintended consequences of increased price, such as compensatory smoking behavior or greater use of contraband cigarettes. Indeed, it is crucial to understand the extent to which compensatory smoking behavior or use of contraband cigarettes may alter the intended impact of public health policies so that interventions can be designed with greater effectiveness.

### 4.1. Limitations of Study

There are limitations in the evidence base of some of the subpopulations. The lack of data makes it difficult to assess the impact of taxation on certain groups or to evaluate trends across the six subpopulations. The majority of studies on youth smoking rely on data from school-based surveys. These surveys do not capture smoking rates of high school dropouts, street and homeless youth. While prevalence rates in this group are often difficult to ascertain, they are thought to be substantially higher than those of the general youth population.

Most studies reviewed are cross-sectional and do not have the methodological rigor of longitudinal research. Also, studies generally have used large datasets that are not built to address specific questions regarding taxation. Since smoking behavior is determined by a number of factors and their interactions, it is a challenge to elucidate the specific impact of price.

### 4.2. Recommendations

The following recommendations address policy and research needs for reducing the disproportionate burden of tobacco use among the six subpopulations (Table 4 gives further details):

- Policy
  1. Increase price of cigarettes through higher cigarette taxes, or by using other similar or complementary means, such as minimum prices.
  2. Implement “smart tax” policies: Maximize the benefits of tobacco taxation by:
    ♦ restricting price-based promotions, and
    ♦ aggressively curbing sale of contraband cigarettes.
- Research
  1. Conduct a meta-analysis of studies reviewed in this report:
    ♦ to provide a robust estimate of the magnitude of price-responsiveness, particularly for youth and young adults.
  2. Study the effects of large tax increases:
    ♦ to determine whether large tax increases have greater or lesser effects than smaller tax increases. Generally smaller changes in cigarette tax increases have been studied to date.
  3. Establish a centralized repository of data:
    ♦ to ensure that datasets can be readily accessed to facilitate research on tobacco control policies, including taxation. This would encourage consistent measures of smoking behavior and price measures.
  4. Examine synergies between taxation and other tobacco control policies:
    ♦ to determine whether there are important synergies between tobacco taxation and other tobacco control policies that may either enhance or moderate effects. Multivariate analyses (e.g., hierarchical linear modeling; path analyses; structural equation modeling) can help elucidate the differential and combined effects of various policies.
  5. Study the unintended consequences of increased taxation on cigarettes:
    ♦ to determine whether inadvertent effects such as price minimization strategies diminish the public health value of tax increases.
  6. Incorporate gender stratification routinely in study designs and analyses:
    ♦ to determine whether there are gender differences in the effects of increased cigarette prices on smoking behavior. While some studies in this review analyzed results by gender, the majority did not.
  7. Expedite research on dual diagnosis, heavy/long-term smokers and Aboriginals:
    ♦ more and better data are needed on these subpopulations because of higher smoking rates and limited research.

## 5. Conclusions

Significant strides have been made in reducing smoking over the past three decades, particularly in high-income countries. Nevertheless, the health toll of smoking remains a compelling global health challenge. Concerted efforts are needed to reach a higher summit in tobacco control, especially with subpopulations at most risk.

The economic literature has made unique and important contributions to our understanding of the effectiveness of tobacco taxation on ameliorating the health consequences of smoking. Increased tobacco taxes, passed on to consumers in the form of higher cigarette prices, provide an economic disincentive to those who smoke or may be contemplating smoking. Indeed, the evidence from this knowledge synthesis strongly supports increasing cigarette prices through tobacco taxation as a powerful strategy for achieving major reductions in smoking behavior among some, but not all, high-risk populations.

For instance, increasing the price of cigarettes is a very effective policy tool for reducing smoking participation and consumption among youth, young adults and persons of low socioeconomic status. In contrast, major gaps exist in our knowledge about the impact of price on persons diagnosed with mental health or non-nicotine substance abuse disorders, heavy and long-term smokers, and Aboriginal people. Given their high prevalence of smoking, urgent attention is needed to develop effective tobacco control policies for these subpopulations. A related issue is whether or not increased prices have an effect on reducing smoking initiation among youth and young adults.

The findings from this study should be of particular value to policy-makers and researchers in their efforts to design and improve the effectiveness of tobacco control measures. Although further work is needed, tobacco taxation is a key policy measure for driving success.

*Benjamin Franklin once said, “In this world, nothing can be said to be certain, except death and taxes.” Yet we have a tax that could prevent hundreds of millions of premature deaths. It is time to use it more effectively* [123].

## Figures and Tables

**Table 1:** Subpopulation size and smoking prevalence (2006–2008) [28–32].

Subpopulation	% of Total population		Smoking prevalence (%)	

	Canada	US	Canada	US
General Population	33,212,696 (total population)	303,824,640 (total population)	19	20.8
Youth	6.8	7.2	15	22
Young Adults	9.2	9.9	25	26
Low SES	11.4	17	**	**
Income *				
Education				
Dual Diagnosis	5–10	5–10	38–57	41–62
Heavy and/or Long-term Smokers	**	**	**	**
Aboriginals	3.8	1.5	60	32

* OECD definition of poverty: population below 50% of median income.

** Information unavailable.

**Table 2:** Impact of increased taxation and price on youth smoking behavior.

Results	Initiation	Cessation	Stages of smoking uptake	Participation (prevalence)	Consumption (quantity smoked by smokers)
Yes, reduces youth smoking	7	5	3	23	17
No, does not reduce youth smoking	9	3	0	4	2
It depends—reduces smoking in some cases	6	2	2	4	1
**Totals**	**22**	**10**	**5**	**31**	**20**

**Table 3:** Impact of increased taxation and price on young adult smoking behavior.

Results	Initiation	Cessation	Stages of smoking uptake	Participation (prevalence)	Consumption (quantity smoked by smokers)
Yes, reduces young adult smoking	1	4	1	9	10
No, does not reduce young adult smoking	3	1	0	1	0
**Total # Studies**	**4**	**5**	**1**	**10**	**10**

**Table 4:** Subpopulation recommendations.

Subpopulation	Recommendations
***Youth*** ***Young Adults***	Conduct further studies on the impact of cigarette price on smoking initiation Evaluate the distinctions and differential impacts of higher cigarette prices on subsets of youth—e.g., by gender, income, school status, age (younger *vs.* older) Conduct research on the importance of other determinants of youth and young adult smoking behavior in addition to cigarette price (e.g., peer and parental influence)
***Low SES***	Combine price increases with a comprehensive tobacco prevention and control program for maximum effectiveness. Accompany increased prices with other tobacco control measures, specific to low SES populations, e.g., expanding/improving smoking cessation resources and providing ways to mitigate hardships due to higher taxes, such as free nicotine replacement therapies.
***Dual Diagnosis*** ***Heavy and/or Long-Term Smokers***	Determine the effectiveness of increasing the price of cigarettes as a policy tool for reducing smoking among these subpopulations.
***Aboriginal People***	Examine the effectiveness of increasing the price of cigarettes as a policy tool for reducing smoking among Aboriginal people. Conduct research on the following topics: impediments to using taxation as a tobacco control policy; effective processes for implementing taxes on tobacco products; impact of taxation and price of cigarettes on smoking behavior of those living on-reserve and off-reserve, and in reducing contraband sales.
